# Development of IgG Mediated Antibody Dependent Cell-mediated Cytotoxicity (ADCC) in the Serum and Genital Mucosa of HIV Seroconverters

**DOI:** 10.4172/2155-6113.1000479

**Published:** 2015-07-09

**Authors:** Mariam Aziz, Fareeha Mahmood, Mariana Mata, Helen G Durkin, Chenglong Liu, Ruth M Greenblatt, Marek Nowicki, Elizabeth T Golub, Kathryn Anastos, Audrey L French, Linda L Baum

**Affiliations:** 1 CORE Center, Cook County Health and Hospitals System, Chicago, IL 60612, USA; 2 Division of Infectious Diseases, Rush University Medical Center, Chicago, IL, 60612, USA; 3 Department of Immunology/Microbiology Rush University Medical Center, Chicago, IL, 60612, USA; 4 SUNY Downstate Medical Center, Brooklyn, NY, 11203, USA; 5 Georgetown University, Washington, DC, 20057, USA; 6 University of California, San Francisco, CA, 94110 USA; 7 University of Southern California Norris Hospital, Los Angeles, CA, 90033 USA; 8 Johns Hopkins University, Baltimore, MD 21218, USA; 9 Montefiore Medical Center, Bronx, NY, 10467 USA

**Keywords:** Women, HIV, Seroconverters, Antibody dependent cellular cytotoxicity

## Abstract

**Background:**

We measured antibody-dependent cell mediated cytotoxicity (ADCC) activity in serum and genital fluids of heterosexually exposed women during HIV seroconversion.

**Methods:**

Plasma and cervico-vaginal lavage (CVL) fluid from 11 seroconverters (SC) were analyzed biannually from one year pre- to 6 year post-seroconversion using a ^51^Cr-release assay to measure HIV-1 gp120 specific ADCC.

**Results:**

No SC had significant HIV specific CVL ADCC activity before seroconversion or until 1.5 yr after seroconversion. One individual had a %Specific Release (SR) of 25.4 at 2 years, 26.7 at 3 years and 21.0 at 4 years after seroconversion in CVL. Another sample had 4.7% SR at 2 years, 5.3 at 3 years, 10.9 at 4 years, and 8.4 at 5 years after seroconversion in CVL. A third had no activity until 17% SR 5 years after seroconversion in CVL. A fourth showed activity of 36.5% SR at 6.5 years after seroconversion. Seven women had no ADCC activity in their CVL. Paired serum samples showed HIV specific ADCC activity prior to the appearance of CVL ADCC activity.

**Conclusions:**

HIV specific ADCC activity in CVL rose 2 years after seroconversion; ADCC was present in the serum prior to this time. These data suggest that genital tract ADCC activity is not present until well after acute infection.

## Background

Attempts to develop a vaccine to prevent HIV have met with minimal success and have stimulated renewed interest in finding alternative ways to generate a protective immune response. There is growing evidence that the HIV-specific antibodies whose activity is mediated through the Fc-receptor, such as antibody dependent cellular cytotoxicity (ADCC), have an important role in controlling HIV infection. ADCC antibodies can link virus-infected cells with effector cells (NK cells, monocytes and neutrophils) that can kill the infected cell and prevent further dissemination of the virus and disease progression. Results from a recent vaccine trial in Thailand, RV144, showed that effectiveness of this vaccine was around 30%. Even though the protection was modest, this trial did show that vaccination can stimulate protective immunity against HIV [1]. Further analysis of the results indicated that non-neutralizing antibodies, including antibodies that mediate ADCC against HIV, contributed significantly to the protection that was observed [2–6]. Studies subsequent to this trial support the protective effect of ADCC antibodies against retroviruses [7–9]. The most convincing of these recent studies shows that vaccination of rhesus macaques with a live attenuated SIV protects against vaginal challenge with a neutralization-resistant SIV strain which correlates with the presence of ADCC antibodies [10]. Additionally, ADCC antibodies in breast milk are associated with reduced risk of mother-to-child transmission [11] and ADCC antibodies exert pressure that leads to generation of viral escape mutants [12].

ADCC protects against intracellular pathogens, including herpes simplex virus, rubella, Epstein-Barr virus, and influenza viruses [1,13–16] and several early studies reported ADCC activity against the HIV-1 envelope glycoprotein, gp120 [17–23]. IgA is often thought of as the primary immunoglobulin subclass at mucosal sites; this generalization does not extend to the response to HIV in the female genital tract. The vast majority of HIV infected women have IgG antibodies against HIV present in vaginal fluids; HIV specific IgA can be detected in less than a quarter of infected women [24,25]. This is an important consideration since HIV specific immunoglobulin in the genital tract of women may be the initial defense against heterosexual transmission. Earlier studies of ADCC in HIV infected women revealed that while almost all of these women had HIV specific serum ADCC antibodies, far fewer, about 60%, had HIV gp120 specific CVL ADCC antibodies [26,27]. A comparison of the presence of antibodies in the CVL of over 108 women from the Division of AIDS Treatment and Research Study 009 (DATRI009) showed that only women who had HIV gp120 specific IgG antibodies in their CVL had IgG mediated ADCC activity [26].

Both systemic and genital tract IgG mediated ADCC may impact HIV infection. Women with HIV specific genital ADCC have lower genital viral loads [26]. ADCC appears to be higher in HIV infected individuals who are able to maintain low plasma HIV RNA levels despite lack of antiretroviral therapy, i.e., elite controllers (EC) [28]. Passively acquired ADCC activity in HIV infected infants was associated with increased survival [29]. Studies have also shown that broader ADCC responses were associated with slower progression of HIV infection [30] and more recently studies have shown that HAART improves HIV specific ADCC in infected individuals [31]. Analysis of sera shortly after HIV-1 infection show that ADCC-directing antibodies are among the earliest to appear [32,33]. These antibodies may have an active role in the control of viremia following early and acute infection [34,35] and may have important implications in the design of future vaccines. We designed this study to measure the development of ADCC activity in serum and genital fluids of women over time. We obtained samples from participants who enrolled in the Women’s Interagency HIV Study (WIHS) when they were seronegative; their risk for seroconversion was heterosexual intercourse, and they seroconverted while still participants in the WIHS. As a result we were able to obtain samples from visits prior to seroconversion and during the six and a half-year period following seroconversion.

## Methods

### Setting and participants

The WIHS is a prospective cohort study of HIV-infected and uninfected at-risk women from six sites: Chicago, IL; the San Francisco Bay Area, CA; Brooklyn, NY; Bronx/Manhattan, NY; Washington, DC; and Los Angeles, CA. Women were seen semiannually for an interview and a physical exam with collection of blood and genital specimens at each visit. Informed consent was obtained from all participants in accordance with the US Department of Health and Human Services guidelines and the Institutional Review Boards of participating institutions. The cohort was designed to reflect the demographics of the HIV epidemic among women in the United States. Details of cohort recruitment, retention and demographics have been published elsewhere [36].

Of the women who experienced HIV seroconversion during the WIHS and had only heterosexual risk, eleven had available serum and cervico-vaginal lavage fluid at pre-seroconversion, seroconversion and post-seroconversion visits for up to 6 years after seroconversion. Concurrent semiannual demographic and clinical data were available on these women as well.

### Laboratory methods

A 4 hour ^51^Cr-release assay with natural killer cells (NK) from healthy known positive donors as effectors was used to measure IgG mediated ADCC against clade specific HIV-1 gp120 coupled target cells. We analyzed plasma and cervicovaginal lavage (CVL) fluid every 6 months for 11 seroconverters (SC) at intervals from one year pre-seroconversion to 6.5 years after seroconversion. Timepoint 0 indicates the visit at which the patient had evidence of seroconversion. We studied from 6 to 9 longitudinal samples for each woman. In total there were 87 visits; 8 of the 11 women had samples from least 8 visits. We evaluated activity for each sample at 4, 10-fold serial dilutions starting with a 1/10 dilution and ending with a 1/10000 dilution. Samples were run in triplicate to determine the mean values with standard deviations. Daily negative controls of NK background cytotoxic activity were done for each assay.

#### Antibody

Serum/plasma or cervical lavage fluid (CVL) from WIHS participants were the source of antibodies for these studies. CVL were collected by WIHS investigators using a standardized procedure where the genital vault is rinsed with 10 milliliters of sterile saline and all fluid is then collected. Cells and cellular debris were removed from CVL by centrifugation. Clinical samples were stored at −80°C. A standardized preparation of pooled IgG from asymptomatic HIV-1 infected individuals (HIVIg; North American Biologicals, Miami, FL) was used as a positive control for ADCC against HIV-1 gp120. Although there is variability in the amount of cervical fluid produced by different women, standardization of the collection procedure and use of longitudinal samples from the same women reduce this variability.

#### ADCC assay

There is not currently one uniformly accepted assay which is used for measuring ADCC. Several different assays are commonly used, all of which yield similar results, but measure different stages of ADCC mediated killing. The assay that is used for these studies, the Cr-release assay, measures target cell lysis. The other most commonly used assay measures the expression of CD107a on the surface of the NK effector cell following activation. We recently published a paper which presents a comparison of these assays in our laboratory and shows that they are valid assays [37]. The Cr-release assay was selected for these studies because of the large volume of samples and antibody dilutions to be measured.

#### Effector cells

Peripheral blood mononuclear cells (PBMC) from the blood of HIV-1 negative donors known to have good ADCC activity in previous assays was collected and cryopreserved. Although ADCC activity varies between normal donors, activity of an individual donor is remarkably stable and good normal donors for ADCC activity are not rare. At any one time 3 to 4 donors were identified who had comparable activity in our assays. PBMC from one of these donors were isolated by Ficoll-Hypaque density gradient centrifugation, washed sequentially in RPMI, PBS + 1% EDTA and RPMI + 10% FBS. Lymphocytes were washed, counted and diluted in RPMI + 10% FBS. Since each donor of NK effector cells can have different baseline ADCC activity, cryopreserved cells are used as effectors for these assays. Effector cells were thawed a day prior to the experiment and incubated overnight with RPMI complete media that did not contain stimulatory cytokines. The day of experiment, the effector cells were washed, counted and used for the assay. These effector cells (50 μl) were added to target cells to yield an effector-to-target (E: T) ratio of 40:1.

Even though NK cells were not purified or enriched for use as effector cells in these assays we will refer to these effector cells as NK cells because they are the only cells in the PBMC population that kill NK susceptible targets (or ADCC targets in the presence of antibody) without antigen priming in a 4 hour assay. T cells require priming and monocyte mediated killing is measured in an overnight assay.

#### Target cells

CEM.NK_R_, a NK cell-resistant clone of the CD4+ T cell line, was incubated with 100μCi of radioactive sodium chromate (^51^Cr) for 1hr at 37o C in a 5% humidified CO_2_ incubator. The cells were washed thoroughly and rotated for 1 hr at RT with 15μg/ml of HIV-1MN (clade B) recombinant gp120 (rgp120). More than ninety percent of these target cells bind rgp120 and background NK cell activity is approximately 5 % Specific Release (%SR). Having clade specific rgp120 on the target was thought to be adequate since previous studies have shown a high degree of cross reactivity even against other clades of HIV gp120 for ADCC.

#### ^51^Cr-release assay

Dilutions of serum and CVL were added to microtiter wells; 1×10^4^ target cells were added and antibody and target cells were incubated for 15min. effector cells were then added to give an E:T ratio of 40:1. Spontaneous and maximum release values were obtained by incubating target cells with buffer or 0.1% Triton X-100 respectively. After 3.5 hr in 5% CO_2_ at 37°C, supernatants were harvested and counted using a gamma counter. Counts per minute (cpm) were converted to %SR using the formula: %SR = [(experimental cpm-spontaneous cpm) / (maximum cpm-spontaneous cpm) × 100.

#### Statistical analysis

Each sample was run in triplicate. NK background control activity was subtracted from each result in order to normalize results. An arbitrary cut off of 5% SR was used to represent a positive result.

## Results

### Demographics

A total of 11 seroconverters were evaluated. Mean age was 43 years (range 28–49). Seven of the eleven (64%) were non-Hispanic African-Americans. Co-morbidities included diabetes mellitus (3/11, 27%), hypertension (2/11, 18%) and hepatitis C (2/11, 18%). No patients reported intravenous drug use. The mean number of male sexual partners in the 6 months prior to pre-seroconversion visit was 1.5 (range 0 – 4). Ten of eleven were current or former smokers. Only 2 patients had self-report of sexually transmitted disease at pre-seroconversion visits (one with genital herpes and one with trichomonas), and 2 self-reported having bacterial vaginosis. Four of the 11 (36%) described having a vaginal discharge or odor at their pre-seroconversion visit. No one had history of cervical cancer at any visit. Six of the 11 patients (55%) were not on antiretroviral therapy during any of the visits evaluated. Four started HAART just prior to the latest visit tested, one of whom was started on a protease inhibitor based regimen.

### ADCC activity at pre-seroconversion visits

**Figure 1:**
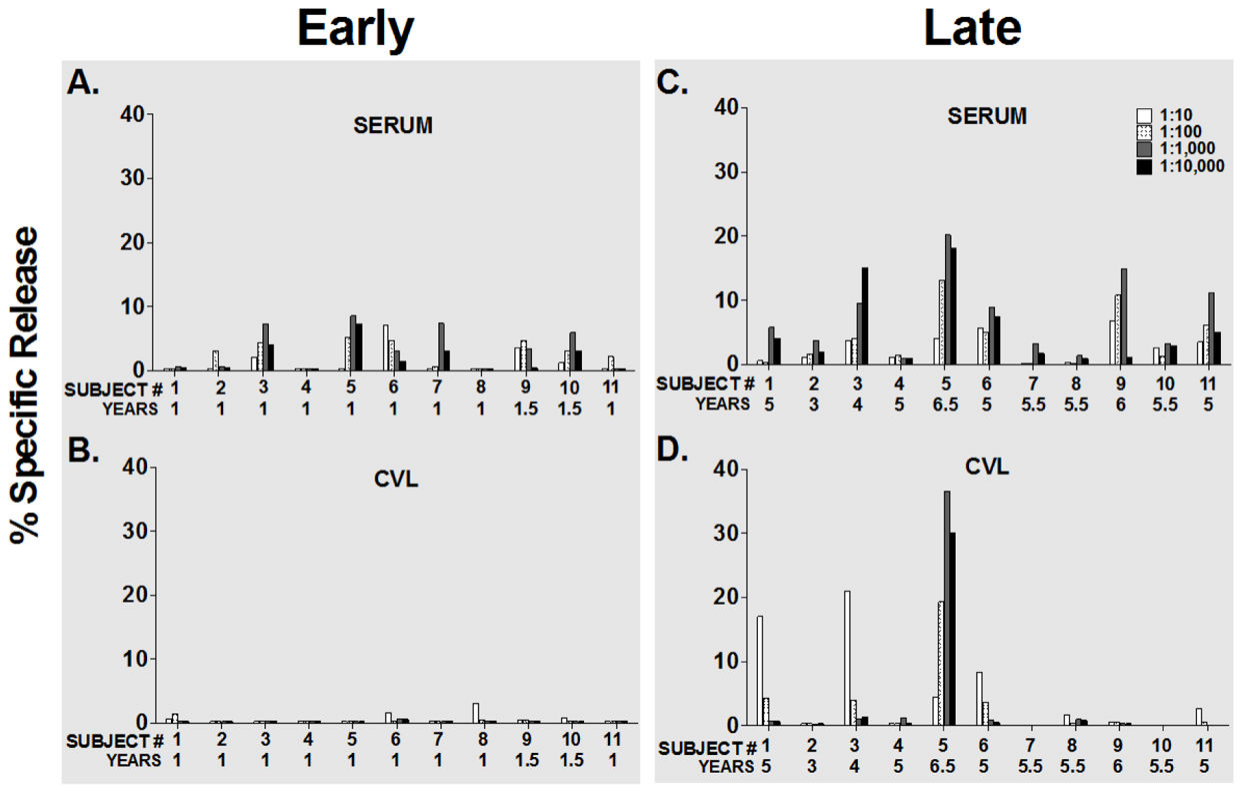
IgG mediated ADCC activity at early and late visits among eleven seroconverters. Paired serum and cervical lavage fluid (CVL) samples were evaluated for ADCC activity against gp120-labeled CEM. NK_R_ at four ten-fold serial dilutions and at a 40:1 effector: target (E: T) ratio in a standard 4-hour ^51^Cr-release assay. Bar graphs represent % specific release (% SR) values after NK (no antibody) background was subtracted. ADCC activity is shown at early visits (<2 years after seroconversion) in (A) Serum samples and (B) CVL samples. On the right panel, ADCC activity is shown at late visits (≥3 years after seroconversion) in (C) Serum samples and (D) CVL samples.

**Figure 2:**
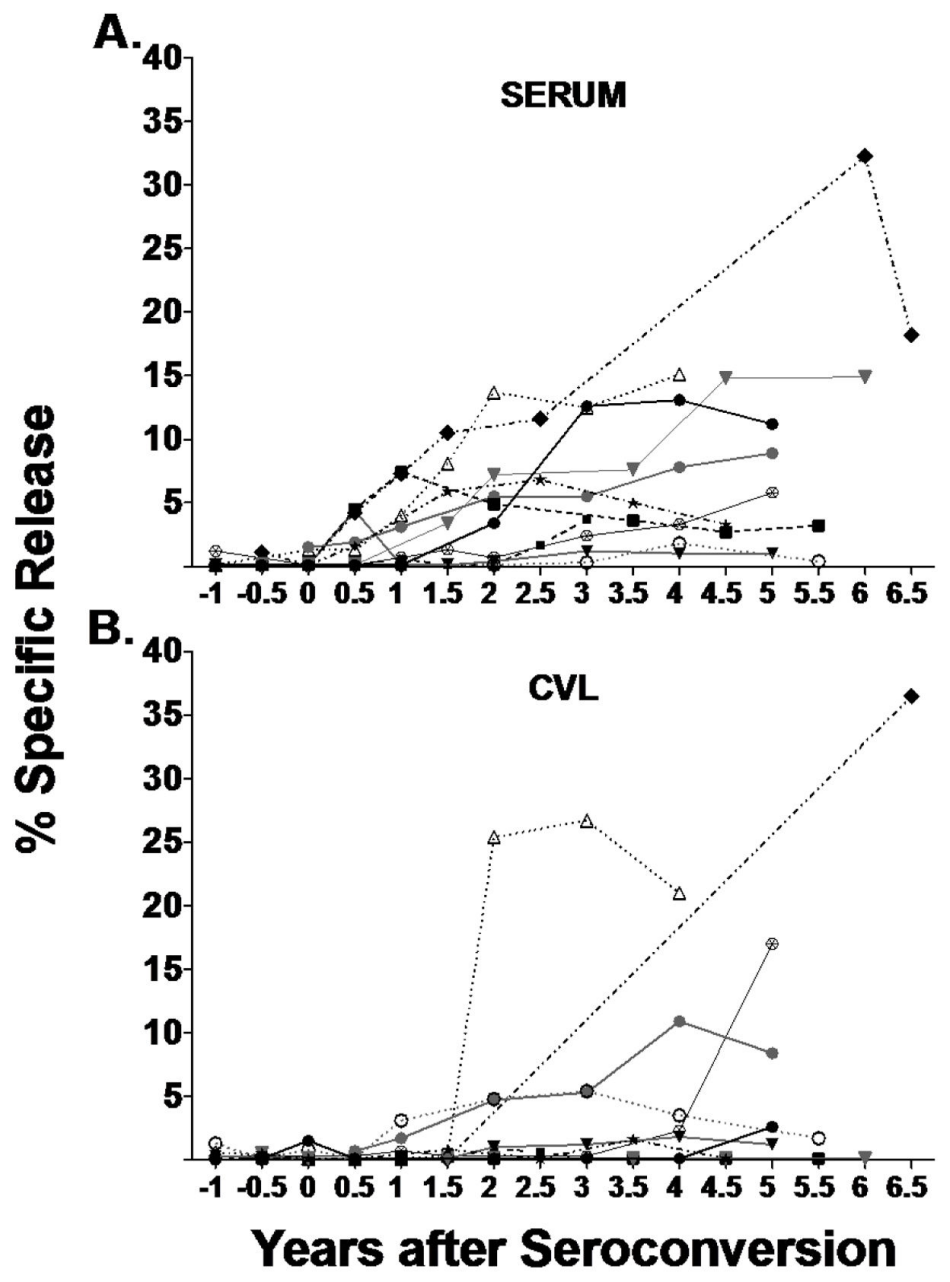
Development of serum and CVL ADCC antibodies following seroconversion. Longitudinal serum and cervical lavage fluid (CVL) samples from 11 individuals were evaluated and compared for ADCC activity over time at their ADCC peak antibody dilution in a standard 4-hr ^51^Cr-release assay. On the X-axis, 0 years indicates time of seroconversion. Plotted symbol/lines represent the % specific release value after NK (no antibody) background was subtracted for each subject. The difference in development of ADCC activity over time is shown in (A) serum samples and (B) paired CVL samples.

There was no HIV specific ADCC activity present in any seroconverter at visits prior to seroconversion. Figures 1A and 1B and 2A and 2B demonstrate this lack of activity for visits one-year prior to seroconversion, 6 months prior to seroconversion and at the visit when seroconversion was diagnosed.

### Comparison of ADCC activity one year or 4–6 years after seroconversion

Figure 1A shows the serum IgG mediated ADCC of the 11 seroconverters at approximately one year after seroconversion. Figure 1C shows CVL IgG mediated ADCC at the same times. There is minimal activity in the serum of all samples, and no activity in the paired CVL samples at this one-year visit. Figure 1B and 1D show IgG mediated ADCC for serum and CVL respectively at later visits (4–6 years after seroconversion). Four of the serum samples at the later time point have IgG antibodies that mediate ADCC that was not present one year after seroconversion. Cervicovaginal lavage IgG activity is also present in 3 of 12 patients at later visits; 4–6 years post seroconversion.

### ADCC activity in serum and CVL of seroconverters at peak dilutions

After seroconversion: Figure 2A shows serum ADCC activity of seroconverters at their peak dilution. Peak dilution is the dilution at which the patient sample had the highest %SR. Figure 2B shows CVL ADCC activity of SC at their peak dilutions. No CVL from seroconverters that we tested had significant ADCC activity against HIV-gp120 from 1 yr before seroconversion to 1.5 yr after seroconversion. One individual had CVL ADCC activity with % Specific Release (SR) +/− 1SD of: 25.4 +/− 0.9 at 2 yr, 26.7+/− 0.7, 3 yr and 21.0 +/− 1.2, 4 yr after seroconversion. Another had: 4.7 +/− 0.9 % SR at 2 yr, 5.3 +/− 0.3 at 3 yr, 10.9 +/− 1.9 at 4 yr, and 8.4 +/− 0.3 at 5 yr after seroconversion in CVL. Subject 1 had no CVL ADCC activity until 5 years after seroconversion, 17 +/− 1.1 %. Subject 5 had no activity around time of seroconversion, but at 6.5 yr after seroconversion had a 36.5 %SR. All subjects had significant serum ADCC activity prior to the appearance of CVL ADCC activity

### Seroconverter ADCC profiles in samples with no activity, activity in serum alone, in CVL alone, or in both

**Figure 3:**
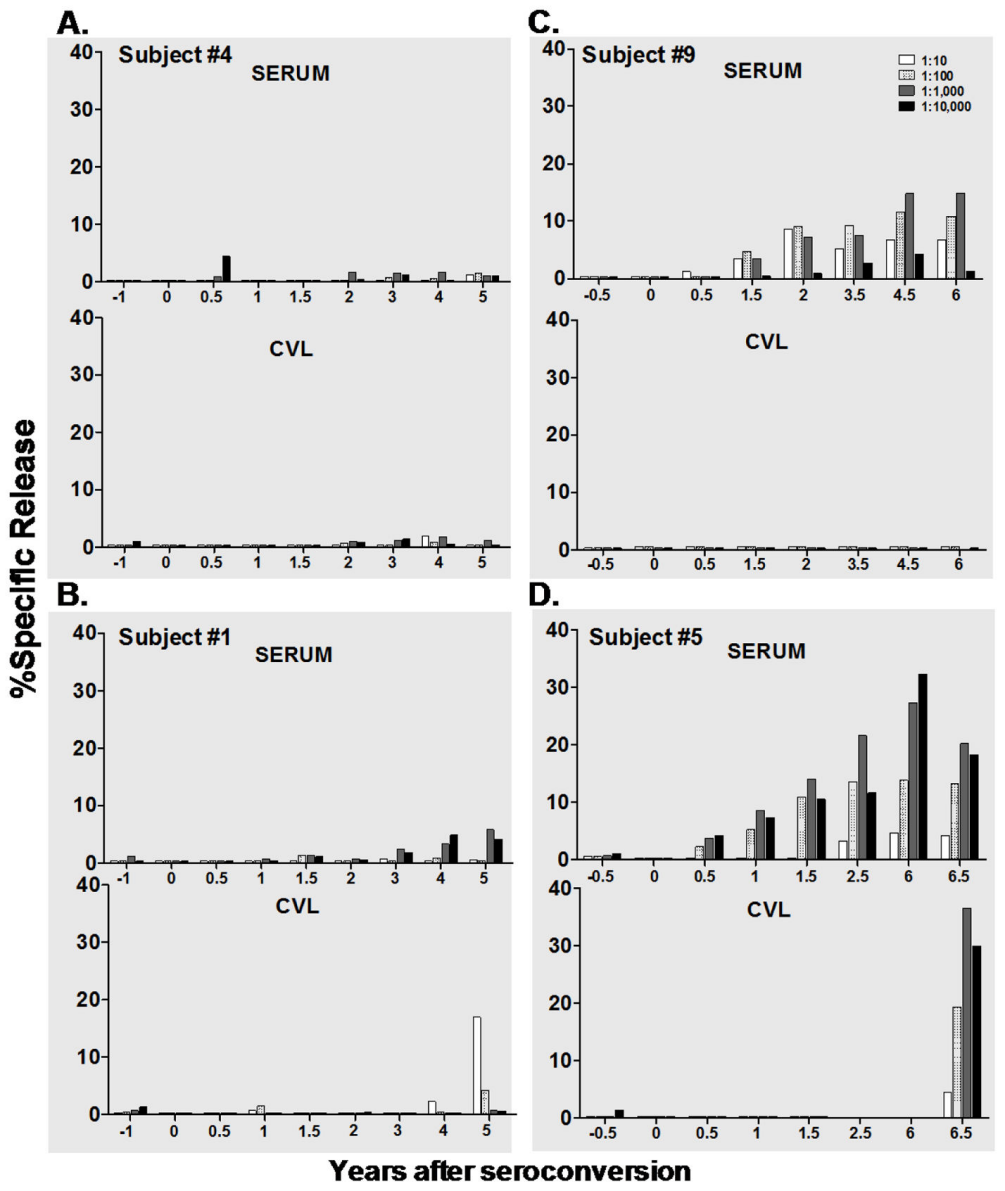
Representative samples illustrating contrasting patterns of development of ADCC antibodies following seroconversion. Paired serum and cervical lavage fluid (CVL) samples were evaluated for ADCC activity against gp120-labeled CEM. NK_R_ at four, ten-fold serial dilutions and at 40:1 effector: target (E:T) ratio in a standard 4-hour ^51^Cr-release assay. Bar graphs represent % specific release (% SR) values after NK (no antibody) background was subtracted. (A) Representative sample with neither serum nor CVL ADCC activity over time. (B) Representative sample showing ADCC activity present in serum only. (C) Representative sample showing ADCC activity acquired over time in CVL only. (D) Representative sample showing ADCC activity in both serum and CVL samples. Note that for subject #5 (in D), no visit samples were available between 2.5 and 6 years after seroconversion.

Figure 3 shows representative serum and CVL ADCC activity at the four dilutions tested: 1:10, 1:100, 1:1000, 1:10,0000. Two of 11 subjects did not have any ADCC activity in serum or in CVL during entire 6 yr follow up (Figure 3A). Four of 11 seroconverters had activity only in the serum (Figure 3C). This representative subject shows a peak serum dilution of 1:1000, and had increasing ADCC activity from 2 years to 6 years, where it peaks at a %SR of 14.9.

Two of the 11 subjects had activity in CVL only (Figure 3B). This representative individual had no activity until 5 years after seroconversion when she had a %SR value of 17 +/− 1.1. Three of the 11 subjects had ADCC activity in both the serum and CVL. One representative individual, (Figure 3D) had a peak serum dilution of 1:1000 with increasing serum activity from 2 to 6 years, with peak %SR values at 6 years of 36.5.

### ADCC activity in a seroconverter with non-specific ADCC activity at seroconversion

**Figure 4:**
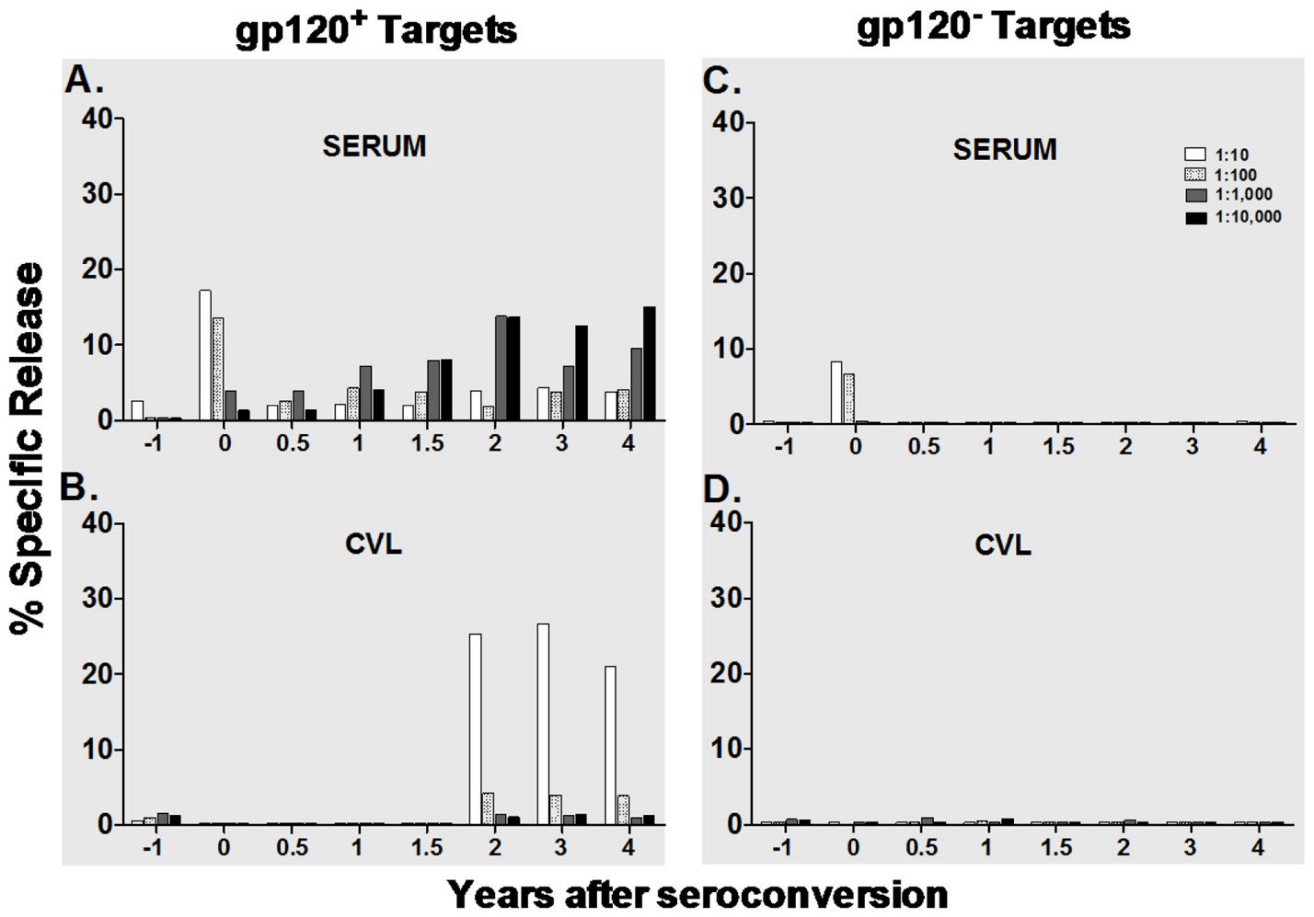
Specificity of ADCC activity in one subject who had unsual serum and CVL ADCC activity at time of seroconversion. Paired serum and cervical lavage fluid (CVL) samples from one subject were evaluated for specificity of ADCC activity at early and late visits following seroconversion. ADCC activity against gp120-labeled or antigen-unlabeled CEM. NK_R_ targets was assessed at four, ten-fold serial dilutions and at a 40:1 effector: target (E:T) ratio in a standard 4-hour ^51^Cr-release assay. Bar graphs represent % specific release (% SR) values after NK (no antibody) background was subtracted. (A) Serovonverter ADCC activity against gp120-labeled targets is shown for serum samples and (B) corresponding CVL samples over time. On the right, ADCC activity against antigen unlabeled-targets is shown for the subject’s (C) serum samples and (D) corresponding CVL samples.

Figure 4 shows a sample from one seroconverter who had significant ADCC activity in the serum at the time of seroconversion (top left) and then increased again around 2 years after seroconversion. The very early appearance of this activity in the serum was unusual because generation of this activity requires specific antigen and time for the development of a primary antibody response. As a result of this, we evaluated the specificity of the activity seen in this individual at all-time points. The antibody present at seroconversion was only found in the serum, this individual did not have any CVL ADCC activity until 2 yr after seroconversion (bottom left). We evaluated the seroconversion samples and compared the activity against our standard target cell, which is coated with recombinant HIV gp120, with uncoated targets (no gp120). The activity, which was present at the time of seroconversion, was nonspecific ADCC activity, rather than HIV specific activity. However, as expected, all of the activity that developed after seroconversion was HIV gp120 specific.

## Discussion

Heterosexual intercourse is the main mode of transmission of HIV worldwide, and HIV specific IgA and IgG in the genital tract of women may be the initial defense against such transmission. The majority of antibodies that mediate HIV specific ADCC activity in the genital tract of HIV infected women are IgG, although IgA is also present [38,39]. These antibodies can link cells that are infected with effector cells (NK cells, monocytes and neutrophils), kill the infected cell and prevent further progression and dissemination of the virus.

Studies from seronegative sex workers in Nairobi, Kenya showed that cervical IgA antibodies played a role in their resistance to infection with HIV [25–28], while another study from highly exposed seronegative women in Cote d’Ivoire, showed that IgG, sIgM and sIgA were present in cervicovaginal lavage fluid and that these antibodies are HIV envelope specific [40]. Previous studies in discordant couples have shown that HIV specific immunity may develop in the absence of infection [29–31].

There is now substantial evidence that ADCC impacts the control of HIV infection. HIV positive men who were long-term survivors from the Multicenter AIDS Cohort Study (MACS) have higher serum ADCC titers than matched rapid progressors [41]. ADCC activity is higher in patients who are elite controllers than in viremic patients [28]. Most HIV infected women studied from the WIHS have serum ADCC antibodies; half also have HIV specific genital tract ADCC antibodies and these women have lower genital tract HIV viral loads [26,27]. A recent study in rhesus macaques showed that ADCC developed over time during persistent infection with live-attenuated simian immunodeficiency virus, and offered protection against re-infection [10,11]. These studies emphasize the potential impact that ADCC may have on the control and possible prevention of infection with HIV.

While there are some data on male seroconverters and ADCC activity prior to the diagnosis of AIDS [41], little is known about when serum ADCC antibodies appear following seroconversion in women. Our results confirm and extend studies of serum ADCC in individuals with early and acute HIV infection [34,35]. The first of these studies reported results from 3 men and 1 woman, all of whom had ADCC against HIV-gp120 at early stages of HIV infection [35]. The second evaluates 8 subjects who controlled viremia to low levels. We are not told the gender of these individuals, but 50% had ADCC antibodies against HIV-gp140 in their serum within the first year; 3 more (a total of 7/8) had activity 2 years after infection [34]. This study differs from ours because we did not select subjects on the basis of their ability to control viral load. None of the published studies describes the timing of appearance of ADCC antibodies in the serum of women, whose major risk factor for infection with HIV is heterosexual sex. Nor are there studies that describe the time course of development of CVL antibodies in the genital tract of women.

Our study suggests that the IgG that mediates ADCC activity in cervico-vaginal lavage fluid does not arise at the time of seroconversion. Our data show that even though seven of the eleven seroconverters had serum activity within the first 2 years following seroconversion, ADCC activity in CVL rose to detectable levels at the earliest, 2 years after seroconversion.

Interestingly, we had one sample that had a high level of ADCC activity at the time of seroconversion, but this activity was not HIV specific. This nonspecific activity was unexpected and may only occur rarely. HIV specific antibodies did not appear as early as we had expected; serum anti-HIV antibodies arose in 8 of the 11 women we studied in the window evaluated. Since nearly all of the women in a cohort of women who were already HIV infected had serum activity and 50% had CVL activity [26], it is reasonable to assume that similar percentages of these women (seroconverters) would develop ADCC activity if they were followed long enough. ADCC activity in the CVL and in the serum may have an active role in the control of viremia following acute infection, and perhaps could protect patients from seroconversion if elicited earlier after exposure. Recent studies have observed ADCC antibody responses and their associated protection, in rhesus macaques elicited by live attenuated SIV, and in the breast milk of HIV-infected mothers whose newborns remain uninfected [10,11].

Since our assay measures ADCC activity against HIV-1 MNgp120, which is CXCR4 tropic, it is possible that these individuals did have ADCC against gp120 from a CCR5 tropic virus or against HIV peptides other than gp120. Evidence that suggests this is not likely to be the case includes: (1) studies that show that much of the ADCC against HIV is against HIV gp120 [42] and (2) studies in our lab and others which indicate that there is a great deal of cross reactivity in ADCC against different clades of HIV gp120 [43,44].

## Conclusions

Our data suggest that local ADCC activity in cervical lavage fluid was not present prior to seroconversion and did not develop until at least 1.5 years after acute infection. This suggests that this potentially important defense mechanism would not be available to an individual who is seeing HIV for the first time. Since innate immune cells can mount an ADCC response against infected targets from another individual, vaccination could be used to induce HIV specific ADCC antibodies that could potentially defend against the initial events of transmission by killing infected cells before maturation of infectious virus within those cells. This hypothesis is supported by studies from non-HIV studies where investigators have shown that ADCC monoclonal antibodies against antigens on cancer cells can be successfully used to treat certain cancers [45,46]. More recent studies showed protection of SHIV and SIV challenged macaques [10,47,48]. This information could inform future vaccine development as the induction of ADCC activity may in the future translate into protection from HIV seroconversion.
